# Molecular investigation of infection sources and transmission chains of brucellosis in Zhejiang, China

**DOI:** 10.1080/22221751.2020.1754137

**Published:** 2020-05-07

**Authors:** Heng Wang, Wei-min Xu, Kuang-ji Zhu, Su-juan Zhu, Hong-fang Zhang, Jia Wang, Yang Yang, Feng-yao Shao, Neng-ming Jiang, Zhen-yang Tao, Hang-yi Jin, Yi Tang, Liang-liang Huo, Fang Dong, Zhen-jun Li, Hua Ding, Zhi-guo Liu

**Affiliations:** a Hangzhou Center for Disease Control and Prevention, Hangzhou, People’s Republic of China; b Jinhua WuCheng District Center for Disease Control and Prevention, WuCheng, People’s Republic of China; c Tongxiang Shi Center for Disease Control and Prevention, Tongxiang, People’s Republic of China; d Jinhua Jindong District Center for Disease Control and Prevention, Jindong, People’s Republic of China; e State Key Laboratory for Infectious Disease Prevention and Control, National Institute for Communicable Disease Control and Prevention, Chinese Center for Disease Control and Prevention, Beijing, People’s Republic of China

**Keywords:** *B. melitensis*, *B. abortus*, MLVA, trace-back, laboratory infection, Zhejiang province

## Abstract

In the present study, a total of 7793 samples from 5 different types of hosts were collected and tested, with a seroprevalence of 2.4% (184/7793). Although the seroprevalence of human and animal brucellosis is relatively low, numbers of human brucellosis cases reported have increased continuously from 2004 to 2018. A total of 118 *Brucella* strains containing 4 biotypes were obtained, including *Brucella melitensis* bv.1 (*n* = 8) and bv.3 (*n* = 106), *Brucella abortus* bv.3 (*n* = 3) and bv.7 (*n* = 1). Twenty-one shared MLVA-16 genotypes, each composed of 2 to 19 strains obtained from different hosts, suggest the occurrence of a brucellosis outbreak epidemic with multiple source points and laboratory infection events. Moreover, 30 shared MLVA-16 genotypes were observed among 59.6% (68/114) *B. melitensis* isolates from Zhejiang and strains from other 21 different provinces, especially northern provinces, China. The analysis highlighted the imported nature of the strains from all over the northern provinces with a dominant part from the developed areas of animal husbandry. These data revealed a potential transmission pattern of brucellosis in this region, due to introduced infected sheep leading to a brucellosis outbreak epidemic, and eventually causing multiple laboratory infection events. It is urgent to strengthen the inspection and quarantine of the introduced animals.

## Abbreviations

RBPT: Rose Bengal plate test

SAT: standard agglutination test

PCR: polymerase chain reaction

MLVA: multiple locus variable-number tandem repeat analysis

HGDI: Hunter–Gaston discrimination index

MST: minimum spanning tree

## Introduction

Brucellosis is the most common zoonotic disease worldwide, with more than 500,000 new cases being reported annually, and a prevalence rate exceeding 10 cases per 100,000 in some countries [1]. The genus *Brucella*, a Gram-negative, facultative intracellular bacteria, infects a wide range of mammals, including domestic and wild animals as well as humans [2,3]. The *Brucella* bacteria infect humans typically through direct contact with infected animals or consumption of unpasteurized and unboiled milk or fresh cheese. Given this dependence on animal reservoirs [4], the veterinarians, farmers, employees of slaughter houses, and meat processing enterprises are the main threatened population [5]. The course of the disease may be acute, sub-acute, or chronic, and lead to serious damage to the physical and mental health of patients, mainly clinical syndrome, including high fever, night sweat, fatigue, joint pain, and headache [6]. Due to lack of proper treatments and reliable diagnosis methods, brucellosis seriously threatens the human health and is a global public health concern [7].

During 2004–2016, a total of 448,479 cases of brucellosis were confirmed in China, depicting a growing trend for an epidemic across all provinces [8]. Zhejiang Province, known as the “land of fish and rice”, is located on the southeast coast of China. *Brucella abortus* Brucellosis was prevalent in limited regions in the 1950s, due to imported cow from other regions. In 1963, the frequency of positive cows of a dairy farm in Hangzhou city was 22.28%. Subsequently, a comprehensive control strategy was launched, including surveillance quarantine, culling the infected herd, and environmental disinfection. The disease has been effectively controlled, and no human or animal brucellosis was reported until mid-1995s. However, sporadic human brucellosis was observed after the 2000s. With the increase of the livestock and the introduction of live sheep, the prevalence of brucellosis in livestock is increasing gradually, and human brucellosis exhibited a gradually increasing trend after 2005 [9]. Serology surveillance of human and animal brucellosis, identification and genotyping of the circulating *Brucella* species are crucial for the prevention and control of the disease [10,11]. Therefore, detailed knowledge of the epidemiological situation has become very important for the assessment of effective prevention and risk factors of public health. The purpose of this research is to better understand the zoonosis situation of *Brucella* in livestock and the human population. A systematic investigation in *Brucellosis* seroprevalence in humans and animals was performed from 2005 to 2015 in Zhejiang Province, China. To further illuminate the molecular characteristics of circulating *Brucella*, serology testing, bacterial isolation, and multiple locus variable-number tandem repeat analysis (MLVA) were also performed.

## Methods

### Data source

The human reported data in this study were extracted from medical cases occurring during 2004–2018. The numbers of human brucellosis cases obtained from CDC of the people in China must be reported to the Chinese CDC through the National Notifiable Disease Surveillance System. The reported human cases must be accompanied by clinical signs and confirmed by serology test (RBPT and SAT) or the isolation of the organism, in accordance with the case definition of the world health organization [12].

### Sample collection and testing

**Figure 1.**
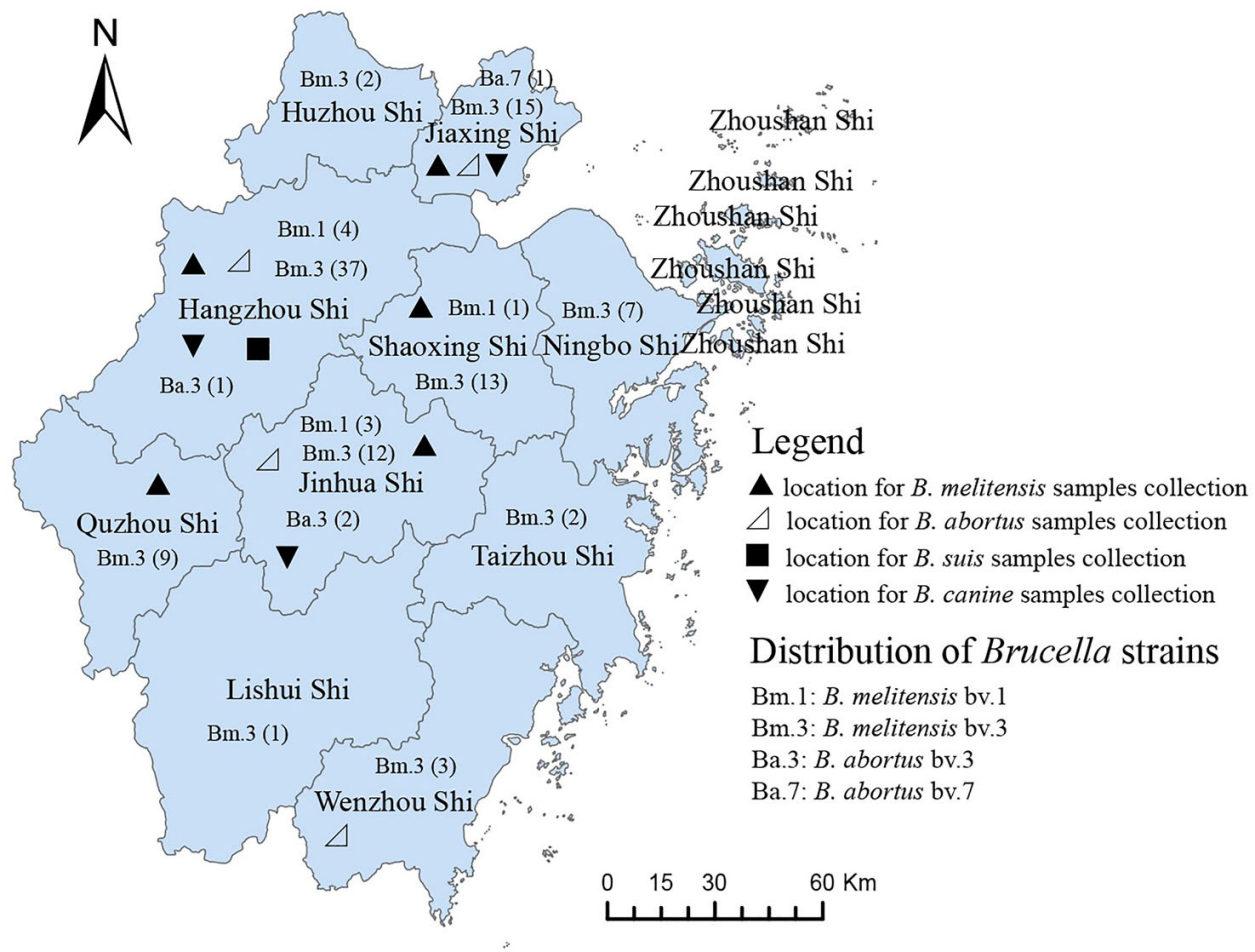
Location for the samples collection and distribution of *Brucella* strains in Zhejiang Province (note: the map does not represent the true borders of administrative regions of Zhejiang, China).

Serum samples and animal products were collected from humans and livestock in the Zhejiang Province, China, from 2006 to 2015. A total of 7793 samples, including humans (2656), sheep (4352), pigs (66), and canines (617), and 102 milk samples were collected from Zhejiang Province, China, of which 1204 were serum samples (292 in breeders, 605 in butchers, and 307 in salesmen) from humans with contact with sheep in seven regions (see Supplementary table 1), 4352 were serum samples of sheep from three regions (see Supplementary table 2), 740 were serum samples (547 in breeder, 42 in milkman, 64 in veterinary of cattle farm, and 87 in salesman) from humans in contact with cattle from four areas (see Supplementary table 3), 624 samples were from canine breeders, and 617 from canines. Eighty-eight serum samples were from pig breeders, and 66 from pigs were also collected (see Supplementary table 4). B*rucella* spp. strain isolation was performed in serum positive samples using a bacteriology method. Moreover, 274 animal products were collected and detected by the bacteriology procedure, including sheep liver, spleen, porcine blood, canine blood, canine liver, duck meat, chicken, and rats from food markets of Zhejiang (see Supplementary table 5). All samples were randomly collected, and the locations of the samples collected are shown in Figure 1. All serum samples were transported to the laboratory, where they were stored at −20°C until processing and detection by the Rose Bengal Plate Test (RBPT). The positive serum samples were reconfirmed by a Standard Agglutination Test (SAT) [13]. Subsequently, bacteriology experiments were performed [14]. All the reagents were purchased from the China Institute of Veterinary Drug Control, where the National Reference Laboratory for Animal Brucellosis is located.

### Isolation of *Brucella* strains

To study the molecular epidemiological characters, all samples with SAT positive were collected and subjected to a brucellosis purification process. The samples were cultured on *Brucella* serum dextrose agar composed of Brucella medium base (supplemented with Brucella selective antibiotic, OXOID, England) and 5–10% heat-inactivated horse serum. The plates were incubated with and without 5–10% carbon dioxide at 37°C after inoculation with sample materials. The plates were examined after 3–30 days for bacterial growth. A single clone was chosen for identification.

### Bacterial strains and convention identification

A total of 118 isolates were isolated from Zhejiang Province from 2006 to 2015. These strains were isolated from human blood, dog, and animal products at the first line laboratory by the Hangzhou Center for Infectious Disease Control and Prevention. One hundred and ten samples were recovered from human blood, 6 from Hu sheep, 3 from sheep, 2 from goat, and 1 from dog. Forty-four strains were isolated from positive surveillance, and others were obtained from clinic brucellosis cases. The examined isolates were identified as *Brucella* species on the basis of morphology and conventional identification methods according to standard biotyping procedures, including requirement of CO_2_ for growth, H_2_S production, sensitivity to thionin (10 and 20 μg/ml), basic fuchsin (20 μg/ml), and agglutination with monospecific antiserum for A and M antigens and phage lysis test (*Tbilisi, Tb; Berkeley, Bk2; Weybridge; Wb*) [14,15]. *B. melitensis* 16M (BM), *B. abortus* 544 (BA), and *B. suis* 1330 (BS) were used as control strains. DNA was isolated using full-automatic nucleic acid extraction system (LLXBIO China Ltd, China) extraction from 48-h cultures according to the manufacturer's instructions. DNA extracted from all isolates was stored at −20°C. Subsequently, all strains were further identified by BCSP31-PCR [16] and AMOS-PCR [17].

### MLVA-16 genotyping

All of isolates were further examined by MLVA, genotyping schedule, and a PCR amplification process as described previously [18,19]. The PCR products were preliminarily evaluated by 2% or 3% agarose gel electrophoresis. Then, positive products were denatured and resolved by capillary electrophoresis on an ABI Prism 3130 automated fluorescent capillary DNA sequencer (Applied Biosystems). Fragments were sized following comparison with a ROX (carboxy-X-rhodamine)-labelled molecular ladder (MapMaker 1000; Bioventures Inc., Murfreesboro, TN, USA) and Gene Mapper software version 4.0 (Applied Biosystems). The fragment sizes were converted to repeat unit numbers using a published allele numbering system [20]. *B. melitensis* bv. 1 16 M was used as control strain to calibrate the VNTR units.

### Analysis of genotyping data

#### Statistical analysis

Throughout the process, Microsoft Excel (Microsoft, Redmond, CA, US) was used for data cleaning. Statistical analysis was performed with SPSS 19.0 (Chicago, IL, USA), *P*-values <0.05 were considered to be statistically significant. Hunter and Gaston diversity index (HGDI) for loci and MLVA panels were calculated to describe discriminatory capacity of each locus [21]. MLVA data were analysed using BioNumerics version 5.1 software (Applied Maths, Belgium). Both categorical coefficient and un-weighted pair group methods with arithmetic mean algorithm (UPGMA) were applied to MLVA clustering analysis (see Table S1). The resultant genotypes were compared using the online *Brucella* 2016 MLVAbank. Minimum spanning tree (MST) based on complete MLVA-16 was used to investigate molecular relationships between strains in this study and 1 344 *B. melitensis* isolates (see Table S2) from other provinces of China (MLVAbank Brucella_4_5) (http://microbesgenotyping.i2bc.paris-saclay.fr/databases/view/1156/).

## Results

### Seroprevalence characteristics of human and animal brucellosis

The incidence of Human brucellosis in Zhejiang Province increased continuously from 2004 to 2018. The incidence of Human brucellosis in 2002 was 0.0043/100,000, increasing to 0.263/100,000 in 2017, i.e. a value is 60 times higher than that in 2002 (see Supplementary figure 1). The seroprevalence of human brucellosis after contact with sheep differed significantly across the seven districts (*χ*^2^ = 28.756, *P* < 0.05), the range of the prevalence of human contact with sheep is 0–100% in the seven regions examined, the average seroprevalence is 5.2% (63/1,204), and 44 *Brucella* strains were obtained from these patients, 22 (50%) of which were from butchers; 20 strains were from Hangzhou, 11 from Tongxiang, 5 from Shangyu city, 3 from the Nanhu district, and 5 from Longyou county (see Supplementary table 1). A total of 4352 blood samples of sheep were tested in the 3 regions examined, and the average positive rate was 1.7% (72/4352). Ten *Brucella* strains were isolated from these samples, of which 7 were obtained from Tongxiang city, and the other 3 from Longyou county (see Supplementary table 2). Seven hundred and forty blood samples from humans contacting the infection from cows in 4 districts were collected and tested. The range of positive rate was 0–11.2%, the average serum positive rate was 5.8% (43/740). Seroprevalence of human brucellosis contracted from cows differed significantly across the four districts (*χ*^2^ = 35.921, *P* < 0.05) and the highest positive rate was observed in the Jindong district (see Supplementary table 3). A total of 624 blood samples of dog owners were tested, and only 1 positive sample was found, and one *Brucella* was isolated from this sample (Published) [22]. Meanwhile, 617 canine blood samples were detected, with a serum positive rate of 0.5% (3/617), and no *Brucella* strain was found (see Supplementary table 4). Moreover, 88 blood samples of pig farmers and 66 blood samples from pigs were collected and tested, which were all negative. A total of 102 raw milk samples were detected, and 2 samples were positive, but no strains were found (see Supplementary table 5). Finally, bacteriology tests were performed in 274 animal product samples, and only one *Brucella* strain was isolated from a liver sample of canine (see Supplementary table 5).

### Characteristics and distributions of isolates

A total of 118 *Brucella* spp. strains were isolated in this study during 2005–2015, among which 118 strains exhibited a convex, circular, and translucent morphology profile. The growth characteristics, phage lysis experiments, dye bacteriostatic tests, and slide agglutination with monospecific anti-*Brucella* sera were used to characterize all isolates (Table 1). Species and biovars were discriminated based on standard bacteriological procedures. Finally, biotyping identified 114 strains as *B. melitensis* (biovar 3 (*n* = 106), biovar 1 (*n* = 8)) and 4 were *B. abortus* (biovar 3 (*n* = 3) and biovar 7 (*n* = 1))*.* A total of 106 strains were isolated from human blood, 6 in Hu Sheep, 3 in sheep, 2 in goats, and 1 in dogs. Except for Zhoushan city, these strains were widely distributed in all 10 other regions of Zhejiang Province (Figure 1), 42 of which were in Hangzhou, 17 in Jinhua city, 16 in Jiaxing city, and 14 in Shaoxing city.

**Table 1.** Biotyping characteristics of *Brucella* species isolates in Zhejiang, China.

Strain No.	Growth characteristics				Monospecific Sera			Phages lysis testing			Interpreted
	CO_2_ requested	H_2_S	BF	TH	A	M	R	Tb	BK_2_	Wb	
BA	+	+	+	−	+	−	−	CL	CL	CL	*B. abortus 544*
BM	−	−	+	+	−	+	−	NL	CL	NL	*B. melitensis16M*
BS	−	++	−	+	+	−	−	NL	CL	CL	*B. suis 1330*
8	−	−	+	+	−	+	−	NL	CL	NL	*B. melitensis bv. 1*
106	−	−	+	+	+	+	−	NL	CL	NL	*B. melitensis bv. 3*
3	−	−	+	+	+	−	−	CL	CL	CL	*B. abortus bv. 3*
1	−	−	+	+	+	+	−	CL	CL	CL	*B. abortus bv. 7*

**Description of data**: Strain No., the number conferred to isolates; BF, Basic fuchsin at 20 μg/ml (1/50,000 w/v); TH, Thionin at 20 μg/ml (1/50,000 w/v); Phages, Tb = Tbilisi, BK_2_ = Berkeley type 2, Wb = Weybridge; CL, Confluent Lysis; NL, No lysis; RTD, Routine test dilution; +, positive (serum agglutination positive); –, negative (serum agglutination negative).

### MLVA genotyping characteristic of *Brucella* isolates

In *B. melitensis* strains, the HGDI value of three loci was >0.7, and the HGDI value of eight loci was <0.1191; the other five loci showed no diversity (HDGI = 0.0000). Moreover, the HGDI value of Panel1, MLVA-11, and MLVA-16 was 0.1667, 0.2141, and 0.9640, respectively (Table 2). In *B. abortus* strains, HGDI of four Panel 2B loci was >0.50, 0.6667 in bruce30, 0.8333 in bruce09, and 0.5000 in bruce55 and bruce18. The other 10 loci showed no diversity (HDGI = 0.0000), the HGDI values of Panel1, MLVA-11, and MLVA-16 were 0.5000, 0.5000, and 0.8333, respectively (Table 2).

**Table 2.** HGDI values of 114 *B. melitensis* isolates and 4 *B. abortus* isolates.

Panels	*B. melitensis* strains (*n* = 114)		*B. abortus* strains (*n* = 4)	
Panel 1	Locus	HGDI^a^	Locus	HGDI^a^
	bruce06	0.0000	bruce06	0.0000
	bruce08	0.0175	bruce08	0.0000
	bruce11	0.0000	bruce11	0.0000
	bruce12	0.0000	bruce12	0.0000
	bruce42	0.0846	bruce42	0.0000
	bruce43	0.0348	bruce43	0.0000
	bruce45	0.0175	bruce45	0.0000
	bruce55	0.0175	bruce55	0.5000
Panel 2A	bruce18	0.0000	bruce18	0.5000
	bruce19	0.0520	bruce19	0.0000
	bruce21	0.0000	bruce21	0.0000
Panel 2B	bruce04	0.7316	bruce04	0.5000
	bruce07	0.0846	bruce07	0.5000
	bruce09	0.1191	bruce09	0.8333
	bruce16	0.7576	bruce16	0.0000
	bruce30	0.7087	bruce30	0.6667
MLVA	Panel1	0.1667	Panel1	0.5000
	MLVA-11	0.2141	MLVA-11	0.5000
	MLVA-16	0.9640	MLVA-16	0.8333

^a^HGDI: Hunter Gaston Diversity Index, which measures the variation of the number of repeats at each locus and ranges from 0.0 (no diversity) to 1.0 (complete diversity).

**Figure 2.**
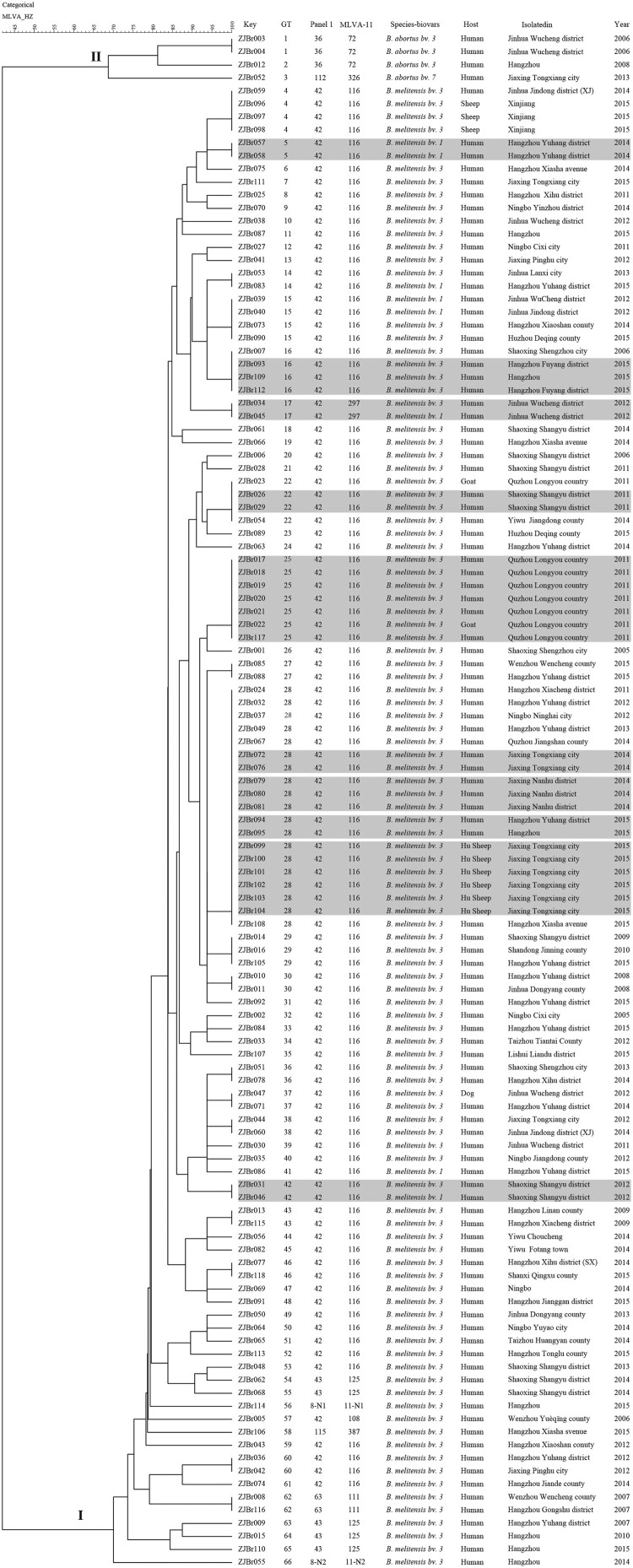
Dendrogram based on the MLVA-16 genotyping assay (UPGMA method), showing the relationships between the 118 *Brucella* isolates. The columns show the identification numbers, MLVA-16 genotypes (GT), panel 1 genotypes, MLVA-11 (panels 1 and 2A) genotypes, species-biovar, host, and the year of isolation of the strains.

Based on the MLVA-16 assay, the 118 strains analysed were sorted into two groups (I and II), four *B. abortus* strains were clustered into group II and formed three MLVA-16 genotypes (GT1–3); the other 114 *B. melitensis* strains were clustered into group I and formed 63 MLVA-16 genotypes (GT4–66) with 70% similarity, of which 20 were shared genotypes that comprised 2–19 strains, and the remaining 43 genotypes were single genotypes, each genotype representing an independent strain (Figure 2). Using panel 1 markers, the present population was clustered into eight panel 1 genotypes, two of them in *B. abortus* strains (36 (1–4–5–3–12–2–2–3–1; *N* = 3) and 112 (3–4–5–3–12–2–2–3–3; *N* = 1)) and six in *B. melitensis* strains,42 (1–5–3–13–2–2–3–2; *N* = 104), 43 (1–5–3–13–3–2–3–2; *N* = 5), 63 (1–5–3–13–2–3–3–2; *N* = 2), 115 (1–4–3–13–2–2–3–2; *N* = 1), N1 (1–5–3–13–2–2–2–2; *N* = 1), N2 (1–5–3–13–2–2–3–1; *N* = 1), and 88.1% (104/118) of isolates belonging to genotype 42 and this panel 1 genotype plays a dominant role in *B. melitensis* (Figure 2). However, 10 MLVA-11 genotypes were identified in these populations, which of 2 MLVA-11 genotypes (72 (*N* = 3) and 326 (*N* = 1)) in *B. abortus* strains, the remaining 8 MLVA-11 genotypes in the *B. melitensis* population*,* including in 116 (*N* = 101), 125 (*N* = 5), 297 (*N* = 2), 111 (*N* = 2), 108 (*N* = 1), 387 (*N* = 1), N1 (*N* = 1), and N2 (*N* = 1), and MLVA-11 genotype 116 accounts for 88.6% (101/114) and is an overwhelmingly predominant population (Figure 2). Subsequently, comparison of these genotypes with MLVAbank suggested that all *B. melitensis* strains of this study belong to the East Mediterranean lineage.

### Genetic relationship analysis of *Brucella abortus* strains

GT1 contained two strains (ZJBr003 and ZJBr004) obtained from two patients from the same farm. The strain had similar MLVA-16 genotypes with strain (2013Jiang#084), which had been collected from Xinjiang, China. ZJBr052 (GT3) was obtained from a patient who was a milkman in a cow farm, and this strain had a genotype similar to that of a strain from Inner Mongolia (2013Jiang#127) (MLVAbank), with three different loci observed among the two strains (see Table S3). ZJBr012 (GT2), which was isolated from a patient who was engaged in beef wholesale for a long time, had a genotype identical to strains (2013Jiang#093) (MLVAbank) from Chongqing, China (see Table S3).

### Investigation of outbreak and laboratory infection of *B. melitensis* strains

Based on MLVA-16, seven shared (GT5, 16, 17, 22, 25, 28, and 42) (Figure 2, genotypes with black shadow) genotypes were from strains found in the same location and period (Figure 2). GT4 contained four *B. melitensis* strains, of which ZJBr059 was isolated from the blood of a human in Hangzhou who worked at sheep farm in Xinjiang Province before onset (Figure 2). GT25 was shared by seven strains, of which ZJBr022 was obtained from a goat in a farm of Longyou county, where an animal brucellosis outbreak had occurred that was caused by goats introduced from Northern China. The other five strains (ZJBr017–021) were from five farm employees, and the remaining strain (ZJBr117) was isolated from the staff of a microbiology laboratory of a hospital in this region. Interestingly, ZJBr017, which was obtained from one of farm worker, was identified as *Brucella* by this staff (Figure 2). GT28 was shared by 19 strains, of which 4 strains (ZJBr076, ZJBr079–ZJBr081) were isolated from four workers in a sheep farm in Jiaxing Tongxiang country, 6 strains (ZJBr099–ZJBr104) were obtained from Hu sheep in this farm, and the remaining 9 strains were isolated from a different region (Figure 2). GT29 was shared by three strains, of which ZJBr016 was isolated from a truck driver who had been trafficking sheep from Shandong Jining county to Hangzhou city many times. The other two strains were from Shaoxing and Hangzhou city (Figure 2). Moreover, GT37 comprised two strains, ZJBr047 and ZJBr071; the former was isolated from dog often fed with sheep offal and the latter from a human (Figure 2). GT46 included ZJBr077 and ZJBr11. ZJBr077 was isolated from a child from Hangzhou city who had drunk raw ewe's milk in grandma's home in Shanxi Qingxu county. ZJBr118 was obtained from a patient in Shanxi Qingxu county (Figure 2). GT43 and GT62 contained two strains each, of which ZJBr013 and ZJBr008 were isolated from the blood sample of two different patients, but ZJBr115 and ZJBr116 were obtained from two clinical laboratory staff members from two different hospitals in Hangzhou city. The two staff members had identified strains ZJBr013 and ZJBr008 without using biosafety protection facilities (Figure 2).

### Molecular epidemiological investigation of 1, 344 Chinese *B. melitensis* strains

**Figure 3.**
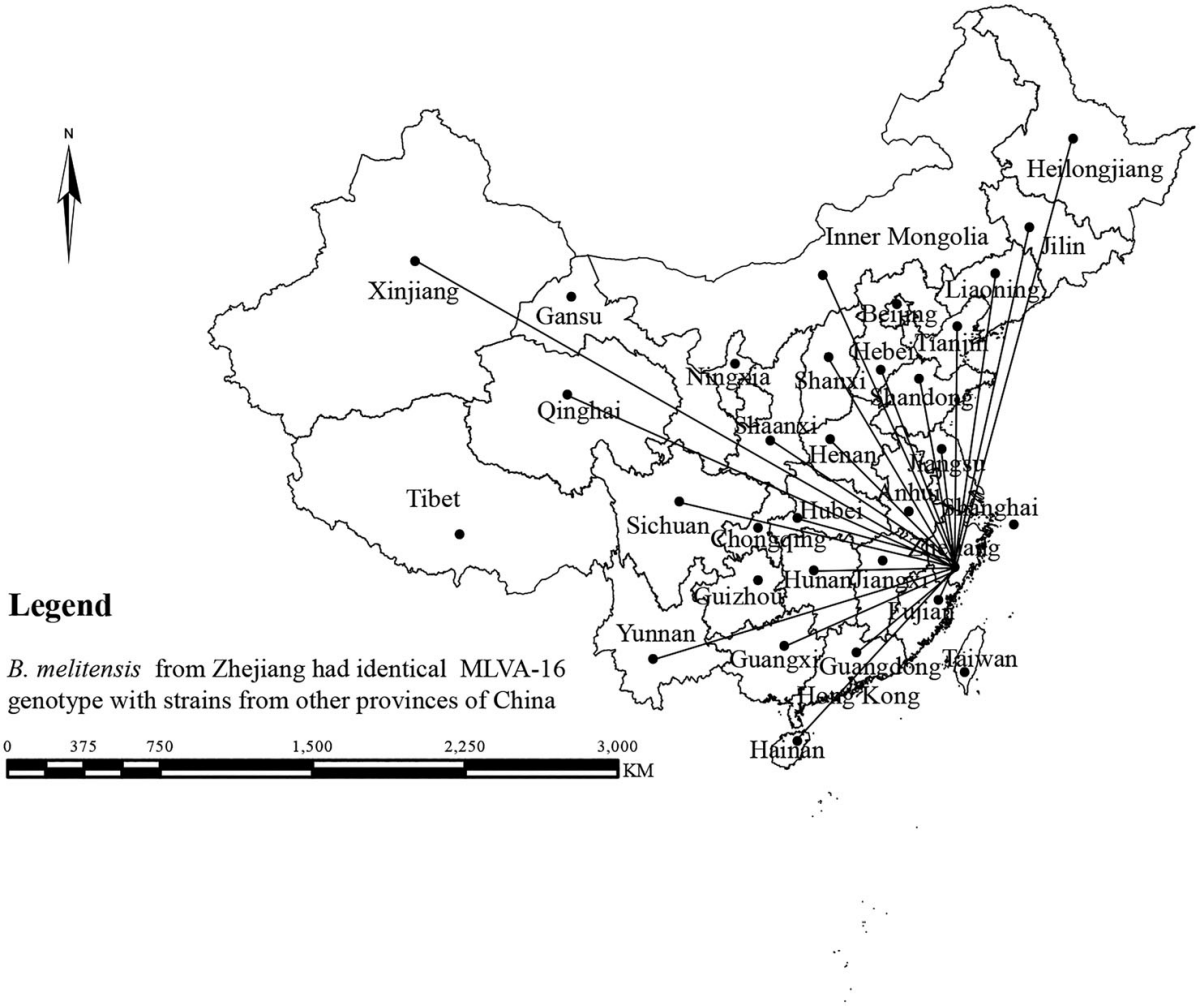
The strain from this study has an MLVA-16 genotype identical to that of strains from 21 different provinces in China (note: the map does not represent the true borders of the administrative regions of China).

**Figure 4.**
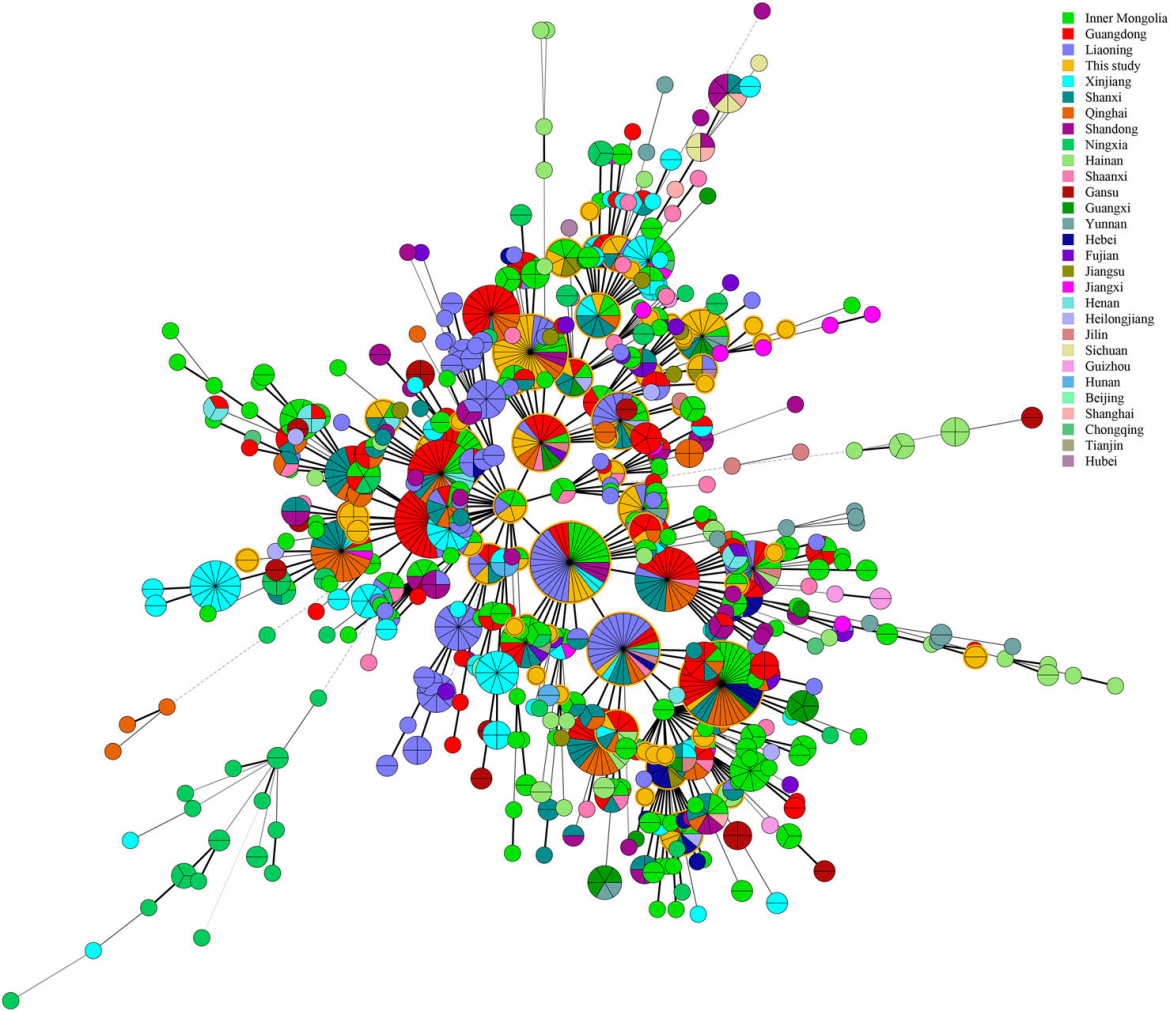
Minimum spanning tree based on MLVA-16 data for *Brucella melitensis* in China (the strains in this study are indicated with yellow circles).

In this study, the MLVA-16 assay was used to investigate molecular relationships between this study's *B. melitensis* isolates and 1344 *B. melitensis* isolates from other provinces of China. Thirty shared genotypes were observed in this population (see Table S4). *B. melitensis* isolates from Zhejiang Province had genotypes identical to those of strains from 21 different provinces (Figure 3), including the Inner Mongolia, Xinjiang, and Qinghai, Shanxi, Heilongjiang, Jilin, Liaoning, Shaanxi, Henan, Hebei, Tianjin, Jiangsu, and Hunan Provinces, i.e. 59.6% (68/114) strains from this study shared MLVA-16 genotypes with strains from these provinces, especially strains from northern provinces (Figure 4). GT28 contained 11 strains from a brucellosis outbreak in a farm. These strains had identical genotype with strains from Inner Mongolia, Liaoning, Qinghai, and Shandong (see Table S4). GT37 comprised two strains from human and dog, which had a genotype identical to that of strains from Shaanxi (see Table S4). GT43 contained two strains from a patient and a laboratorian who had a genotype identical to strains from Inner Mongolia and Jiangsu Province (see Table S4). GT46 contained two strains obtained from a suspect infection event caused by raw ewe's milk. These strains have a genotype identical to that of strains from many northern provinces, including Liaoning, Qinghai, Shanxi, and Tianjin Provinces (see Table S4).

## Discussion

Brucellosis is a common zoonotic disease of a public health menace that is still endemic in many countries and regions, including Zhejiang Province, China [23]. In this study, the seroprevalence of animal and human brucellosis was investigated in many regions, and we performed a phenotypic and molecular characterization of *Brucella* isolates from sheep, Hu sheep, goats, and human samples obtained from different geographical locations of Zhejiang Province. Human brucellosis case reports were rare in Zhejiang Province before the 2000s; however, the incidence rate of human brucellosis has increased continuously during 2004–2018; notably, the incidence rate in 2017 was 60 times higher than in 2002. The epidemic trend of human brucellosis in this region coincides with other southern provinces [9]. The main reason is that people are starting to promote livestock activities, especially sheep breeding in these regions. However, the livestock bred in these regions were introduced almost exclusively from Northern China, where animal husbandry has developed, and a prevalence of more than 90% brucellosis has been reported [24]. The rising incidence rate in this region could have a potential correlation to the trans-boundary transfer of infected animals from Northern China [25].

The average serum positive rate in humans contacting with sheep is 5.2% (63/1204), which is higher than the positive rate in sheep (1.7% (72/4352)). Interestingly, of the 44 *Brucella* strains obtained from these patients, 22 (around 50%) were from butchers, which suggests that butchers are a at high risk of infection. The Zhejiang Province is an economically developed region and numbers of livestock (small ruminate) is less than northern, China, and there is a great demand for mutton consumption. Thus thousands of live sheep have been introduced and slaughtered, and this is a reasonable explanation for the observation that seroprevalence in humans (butchers) contacting with sheep is higher than in sheep. The zoonotic risk of brucellosis posed by *Brucella*-infected slaughtered animals to abattoir workers cannot be ignored in emerging or non-epidemic regions of brucellosis [26]. Moreover, the highest positive rate (28.6%) of sheep brucellosis was found in a farm of Longyou county, and a scene survey showed that 200 Boer goats from Shandong Province had been introduced in this farm. The introduction of infected animals is a main risk factor for the outbreak of brucellosis in sheep herds [27].

The average serum positive rate of human infections contracted from cow was 5.8% (43/740), and the highest positive rate in a human population was observed in the Jindong district, home of the largest dairy farming base of Southern China and a district in which the dairy industry is the main economic source in the rural areas. This conclusion is in agreement with studies reporting that the seroprevalence of dairy cattle brucellosis in Southern China reached 5.5%; however, in Northern China, where the traditional agropastoral areas with the most developed animal breeding industry are located, the dairy cattle seroprevalence was >10% [28]. Our study showed that the serum positive rate of dog owners and dogs in this region was ≤ 0.5%. Although *B. canis* has limited epidemiological significance for the human populations, it remains a significant threat to the canine breeding industry and to humans who come into close contact with dogs [22, 29]. Moreover, no positive samples were observed in pig farmers and pigs, suggesting that the prevalence of brucellosis in pigs is low, but further investigation is needed. Moreover, not only there were few positive samples found in raw milk and animal products, but also one *Brucella* strain was isolated from a liver sample of canine (that often ate sheep viscus), which is a kind of food in local communities. These results revealed that consuming unpasteurized milk and unproperly cooked animal products is a significant public health concern in this region [30].

In the present study, a total of 118 *Brucella* spp*.* strains were isolated, of which 55 strains were obtained from active surveillance, and the remaining 63 strains were isolated from diagnosis of suspect patients. *Brucella* strains were widely distributed in 10 regions (a total of 11 cities), at least 4 species/biovars were found in human and animal hosts, and *B. melitensis* bv.3 was the predominant species. These data indicated that brucellosis in this province is becoming a serious health problem. Both *B. abortus* bv. 3 and bv.7 were first isolated from human contact with cow and beef in China. Although these strains cause infections less severe than those caused by *B. melitensis* or *B. suis* [31,32], they remain an important public health threat to human populations.

MLVA-16 assay displayed high resolution to 114 *B. melitensis* strains, with HGDI value of 0.9640, of which the three loci (bruce04, bruce16, and bruce30) from panel 2B were the most useful for genotyping analysis of isolates from Zhejiang. Based on panel1, two dominated genotypes in *B. abortus* and *B. melitensis,* 36 and 42, are common in Northern China [25]. Subsequently, four *B. abortus* strains were divided into two MLVA-11 genotypes (72 (*N* = 3) and 326 (*N* = 1)) revealed that the *B. abortus* strains in this study are abortus C group descent [33], which shares the geographic origin with strains from Chongqing, Xinjiang, and Inner Mongolia. All *B. melitensis* were clustered with eight MLVA-11 genotypes and belong to the East Mediterranean lineage, of which the MLVA-11 genotype 116 is the overwhelmingly predominant population. Strains belonging to this genotype have an important epidemiology for the human population [34]; these data are in agreement with serious situation of brucellosis in this province.

Based on the comparison of genetic similarly by MLVA-16, three *B. abortus* strains in this study had a MLVA-16 genotype similar to that of strains from developed areas of animal husbandry of Northern China, including Xinjiang and Inner Mongolia. Notably, ZJBr012 (GT2), found in this study, had MLVA-16 genotype completely matching that of a strain from Chongqing, China, historically an epidemic area of *B. abortus* brucellosis [32], suggesting that there are potentially molecular epidemiology links between *B. abortus* strains from this study and strains from Chongqing, Xinjiang, and Inner Mongolia, which may be the origin of the strains we detected. Certainly, genome analysis in these strains is essential to confirm their origin.

Based on MLVA-16 cluster analysis, seven shared genotypes (GT5, 16, 17, 22, 25, 28, and 42) (Figure 2, genotypes with black shadow) consisted of strains from the same location and period, suggesting the occurrence of a multipoint outbreak epidemic from multiple common sources [20]. GT4 contained four *B. melitensis* strains, of which ZJBr059 was isolated from the blood of a human in Hangzhou who worked at a sheep farm in Xinjiang Province before onset, and other three strains were from sheep in this farm, probably the source of infection of this patient from Xinjiang. GT25 was composed of seven strains, of which ZJBr022 was obtained from a goat from a goat farm in Longyou county, where an animal brucellosis outbreak had occurred that was introduced by goats from Northern China. The other five strains (ZJBr017–021) were from workers of this farm, and the remaining strain (ZJBr117) was isolated from staff of microbiology laboratory of hospital in this region. Interestingly, ZJBr017 was obtained from a farmer that was identified by this staff as being infected by *Brucella*. These data confirmed that the introduced goats from Northern provinces led to the brucellosis outbreak of Longyou county farm, and then caused laboratory infection events [35,36]. GT28 was shared by 19 strains, of which 4 strains (ZJBr076, ZJBr079–ZJBr081) were isolated from workers in a sheep farm in Jiaxing Tongxiang country, 6 strains (ZJBr099–ZJBr104) were obtained from Hu sheep in this farm, and the remaining 9 strains were isolated from a different region, suggesting an outbreak of animal and human brucellosis had started from a common source of infection [37]. Interestingly, strains from GT 28 had a genotype identical to that of strains from Inner Mongolia, Liaoning, Qinghai, and Shandong, suggesting a common source of infection outbreak in the farms from these provinces. This conclusion coincides with a field epidemiology survey, as it was found that more than 200 goats from northern provinces had been introduced into this farm. GT29 was composed of three strains, of which ZJBr016 was isolated from a truck driver who was trafficking sheep from Shandong Jining county to Hangzhou city, and the other two strains from Shaoxing and Hangzhou city, suggesting that Jining of Shandong Province is a potential source of infection for these cases. Moreover, GT37 consisted of two strains ZJBr047 and ZJBr071; the former was isolated from dog that was often fed with sheep offal and the latter from humans, suggesting that the two cases shared the source of infection [38]. However, strains from GT37 had a genotype identical to that of strains from Shaanxi, where they may have originated.

GT46 was composed of two strains, ZJBr077 and ZJBr118. ZJBr077 was isolated from a child in Hangzhou city that drank raw ewe's milk in grandma's home in Shanxi Qingxu county, whereas ZJBr118 was obtained from a patient in Shanxi Qingxu county. This result suggests that the source of infection of the child brucellosis was from Shanxi, a region where brucellosis is epidemic [39]. Indeed, strains from GT46 had a genotype identical to that of strains from many northern Provinces, including Liaoning, Qinghai, Shanxi, and Tianjin.

GT43 and GT62 contained two strains each and shared a genotype identical to that of MLVA-16. Two laboratory workers identified strains without using biosafety protection facilities, so these data indicated laboratory infection events [40,41]. Surprisingly, GT43 contained two strains, ZJBr013 and ZJBr115, which were obtained from a patient and laboratorian, respectively, and had the same genotype of strains from Inner Mongolia and Jiangsu Province, suggesting that the source of infection of these events maybe from Inner Mongolia [40, 42]. Because brucellosis is uncommon in Zhejiang Province and patients often present with nonspecific signs and symptoms, clinicians may not suspect brucellosis [43]. Brucellosis is one of the most commonly reported laboratory-acquired infections. The organism is easily aerosolized and has a low infectious dose [35, 44]. Also, laboratory workers may not be familiar with the strains, which can lead to exposures to *Brucella* spp. in clinical laboratories during culturing and isolation of clinical specimens. Working in microbiology laboratories and a lack of compliance with personal protective equipment and biosafety cabinets were the independent risk factors for the development of laboratory-acquired brucellosis. Increased adherence to personal protective equipment and use of biosafety cabinets should be priority targets to prevent laboratory-acquired brucellosis [45].

The 43 single MLVA-16 genotypes suggest that more than 36% (43/118) brucellosis cases are epidemiologically unrelated or sporadic. Moreover, 30 shared MLVA-16 genotypes were observed among 59.6% (68/114) *B. melitensis* isolates from Zhejiang and strains from 21 different provinces, especially northern provinces, China. The analysis highlighted the imported nature of the strains from all over the northern provinces, with a dominant part from the developed areas of animal husbandry. Most imported cases were associated with importation of infected animals. Travel or consumption of unpasteurized dairy products in endemic countries also occurred [46]. We consider that the Zhejiang Province imported animals from regions where brucellosis is epidemic, causing a communication chain leading to a serious brucellosis epidemic and laboratory infection events.

Our study has some limitations, such as the variability in the sample number of serum and strains collected among different regions, and the limited number of strains from animals that were analysed. Whole Genome Sequencing for tracing the geographical origin of the imported cases of human brucellosis is warranted.

## Conclusion

In the present study, 7793 serum samples were tested to anti-*Brucella*, and we found a seropositive rate of 2.4%. A total of 118 *Brucella* spp. derived from humans and animals in Zhejiang from 2005 to 2015 were characterized by classical biotyping and MLVA. Although the numbers of human brucellosis cases reported have increased continuously from 2004 to 2018, the seroprevalence of human and animal brucellosis in this province is relatively low. Classical biotyping revealed the presence of at least four species and biovars. *B. melitensis* was obtained from dogs, sheep blood (food), and laboratory staff members, suggesting that *B. melitensis* is a crucial threat to the occupational population. The *B. abortus* bv.3 and bv.7 were first isolated from humans, and these strains had molecular epidemiology links with strains from Chongqing, Xinjiang, and Inner Mongolia. Circulating isolates were mainly *B. melitensis*, most of them belonging to biovar 3, which is the most abundant biovar in the Mediterranean region. MLVA genotyping confirmed the occurrence of multiple point outbreaks of human brucellosis and laboratory infection events caused by strains imported from Northern China. Our research provides a model for surveillance and control of animals and human brucellosis in other southern regions.

## Declarations

### Ethical approval and consent to participate

This study involves the investigation and isolation of *Brucella* spp*.* using serology, bacteriology, and molecular typing methods. All experiments in the study were approved by the Ethics Committee of the Hangzhou Center for Disease Control and Prevention, and were conducted strictly according to the guidelines. All patients gave written informed consent before participation and all animal samples used in this study were approved by the scientific ethical committee of our institute.

### Consent to publish

Not applicable.

### Availability of data and materials

All data generated or analysed during this study are included in this published article, and its supplementary information files will be freely available to any scientist wishing to use them for non-commercial purposes upon request via e-mail with the corresponding author.

## Supplementary Material

Supplemental Material
