# Supplementary material for: Molecular investigation of infection sources and transmission chains of brucellosis in Zhejiang, China
Source: Emerg Microbes Infect. 2020 May 7;9(1):889–99. doi: 10.1080/22221751.2020.1754137 (PMC7241503; doi:10.1080/22221751.2020.1754137)
Supplement: Supplemental Material [file TEMI_A_1754137_SM0954.zip › Supplementary table1-5.docx]

Supplementary table 1. Seroprevalence of human brucellosis contacted with sheep in seven districts.

| Regions | Samples No. | RBPT | SAT | Positive rate (%) | Strains No. |
| --- | --- | --- | --- | --- | --- |
| Jindong district | 57 | 1 | 1 | 1.8% | 0 |
| WuCheng District | 29 | 0 | 0 | 0 | 0 |
| Hangzhou city | 366 | 20 | 20 | 5.5% | 20 |
| Tongxiang city | 272 | 14 | 13 | 4.8% | 11 |
| Nanhu district | 3 | 3 | 3 | 100% | 3 |
| Shangyu city | 434 | 18 | 18 | 4.1% | 5 |
| Longyou county | 43 | 7 | 7 | 16.3% | 5 |
| Total | 1,204 | 63 | 63 | 5.2% | 44 |

Supplementary table 2. Seroprevalence of sheep brucellosis and *Brucella* isolated in three districts.

| Regions | Sample No. | RPBT | SAT | Positive rate (%) | Strains |
| --- | --- | --- | --- | --- | --- |
| Tongxiang city (Hu sheep) | 4,300 | 60 | 60 | 1.4% | 7 |
| Longyou county (Goat) | 42 | 12 | 12 | 28.6% | 3 |
| Yuhang district (Sheep) | 10 | 0 | 0 | 0 | 0 |
| Total | 4,352 | 72 | 72 | 1.7% | 10 |

Supplementary table 3. Seroprevalence of human brucellosis contracted from cows in four districts.

| Regions | Samples | RBPT | SAT | Positive rate (%) |
| --- | --- | --- | --- | --- |
| Jindong district | 321 | 36 | 36 | 11.2% |
| Wencheng district | 211 | 7 | 7 | 3.3% |
| Hangzhou city | 197 | 0 | 0 | 0 |
| Tongxiang city | 11 | 0 | 0 | 0 |
| Total | 740 | 43 | 43 | 5.8% |

Supplementary table 4. Seroprevalence of canine brucellosis between human and canine in three districts.

| Regions | Human population | | | Canine | | |
| --- | --- | --- | --- | --- | --- | --- |
|  | Samples No. | Positive No. | Strains No. | Samples No. | Positive No. | Strains No. |
| Hangzhou city | 602 | 0 | 0 | 604 | 1 | 0 |
| Jinhua city | 1 | 0 | 0 | 1 | 1 | 0 |
| Jiaxing city | 21 | 1 | 1 | 12 | 1 | 0 |
| Total | 624 | 1 | 1 | 617 | 3 | 0 |

Supplementary table 5. Seroprevalence of brucellosis in animal products.

| Samples | Milk ring test | | Bacteriology | |
| --- | --- | --- | --- | --- |
|  | Numbers | Positive No. | Numbers | Positive No. |
| Raw milk | 102 | 2 | 2 | 0 |
| Lamb (Before storage) | / | / | 100 | 0 |
| Liver | / | / | 60 | 0 |
| Spleen | / | / | 76 | 0 |
| Liver of canine | / | / | 1 | 1 |
| Duck | / | / | 10 | 0 |
| Chicken | / | / | 5 | 0 |
| Rate | / | / | 20 | 0 |
| Total | 102 | 2 | 274 | 1 |
