# Supplementary figures and images for: Molecular investigation of infection sources and transmission chains of brucellosis in Zhejiang, China
Source: Emerg Microbes Infect. 2020 May 7;9(1):889–99. doi: 10.1080/22221751.2020.1754137 (PMC7241503; doi:10.1080/22221751.2020.1754137)

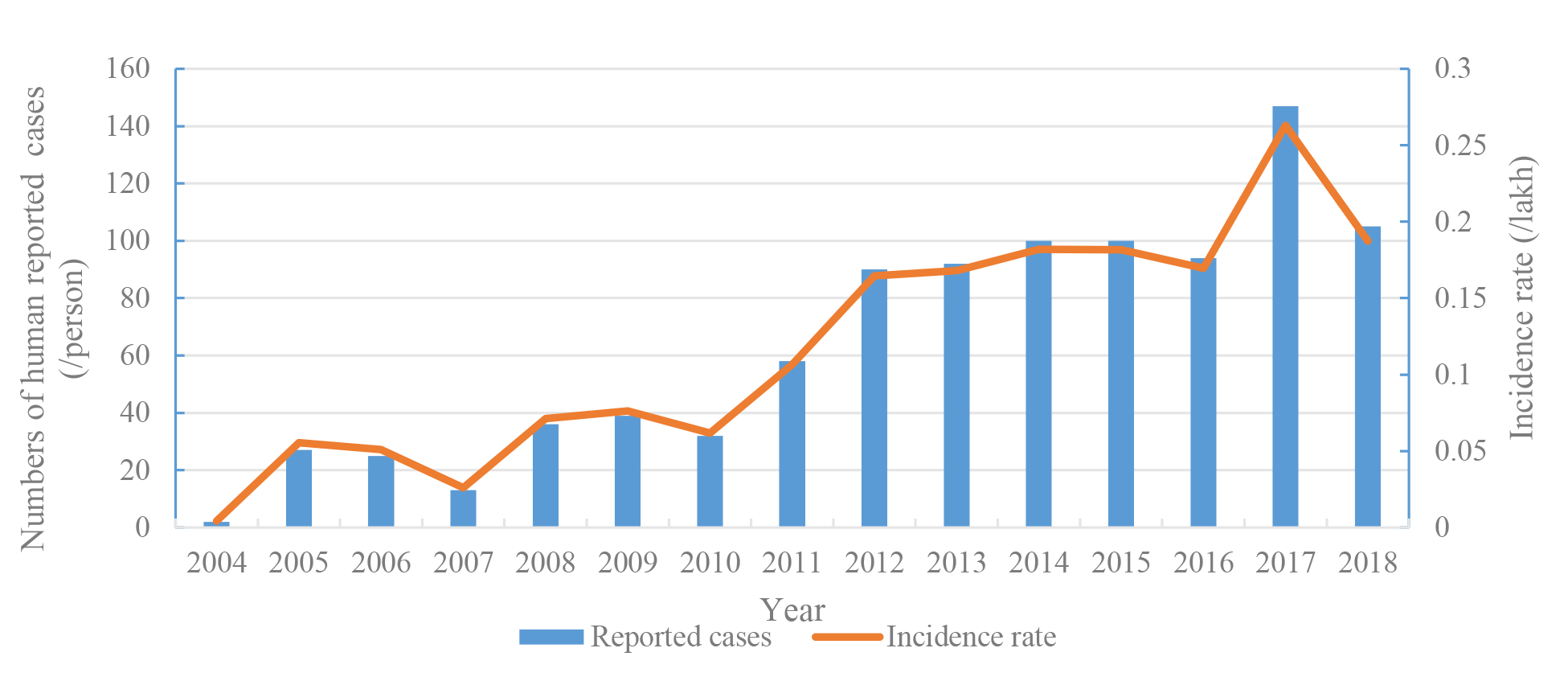

Supplement: Supplemental Material [file TEMI_A_1754137_SM0954.zip › Supplementary fig. 1.tif]
